# Cross-validation of survival associated biomarkers in gastric cancer using transcriptomic data of 1,065 patients

**DOI:** 10.18632/oncotarget.10337

**Published:** 2016-06-30

**Authors:** A. Marcell Szász, András Lánczky, Ádám Nagy, Susann Förster, Kim Hark, Jeffrey E. Green, Alex Boussioutas, Rita Busuttil, András Szabó, Balázs Győrffy

**Affiliations:** ^1^ MTA-TTK Lendület Cancer Biomarker Research Group, Budapest, Hungary; ^2^ 2nd Department of Pathology, Semmelweis University, Budapest, Hungary; ^3^ Max Delbrück Center for Molecular Medicine, Berlin, Germany; ^4^ Transgenic Oncogenesis and Genomics Section, Laboratory of Cancer Biology and Genetics, National Cancer Institute, Bethesda, Maryland, USA; ^5^ Cancer Genetics and Genomics Laboratory, Peter MacCallum Cancer Centre, East Melbourne, Australia; ^6^ Sir Peter MacCallum Department of Oncology, The University of Melbourne, Parkville, Australia; ^7^ Department of Medicine, Royal Melbourne Hospital, The University of Melbourne, Melbourne, Australia; ^8^ 2nd Department of Pediatrics, Semmelweis University, Budapest, Hungary

**Keywords:** gastric cancer, survival, meta-analysis

## Abstract

**Introduction:**

Multiple gene expression based prognostic biomarkers have been repeatedly identified in gastric carcinoma. However, without confirmation in an independent validation study, their clinical utility is limited. Our goal was to establish a robust database enabling the swift validation of previous and future gastric cancer survival biomarker candidates.

**Results:**

The entire database incorporates 1,065 gastric carcinoma samples, gene expression data. Out of 29 established markers, higher expression of BECN1 (HR = 0.68, *p* = 1.5E-05), CASP3 (HR = 0.5, *p* = 6E-14), COX2 (HR = 0.72, *p* = 0.0013), CTGF (HR = 0.72, *p* = 0.00051), CTNNB1 (HR = 0.47, *p* = 4.3E-15), MET (HR = 0.63, *p* = 1.3E-05), and SIRT1 (HR = 0.64, *p* = 2.2E-07) correlated to longer OS. Higher expression of BIRC5 (HR = 1.45, *p* = 1E-04), CNTN1 (HR = 1.44, *p* = 3.5E- 05), EGFR (HR = 1.86, *p* = 8.5E-11), ERCC1 (HR = 1.36, *p* = 0.0012), HER2 (HR = 1.41, *p* = 0.00011), MMP2 (HR = 1.78, *p* = 2.6E-09), PFKB4 (HR = 1.56, *p* = 3.2E-07), SPHK1 (HR = 1.61, *p* = 3.1E-06), SP1 (HR = 1.45, *p* = 1.6E-05), TIMP1 (HR = 1.92, *p* = 2.2E- 10) and VEGF (HR = 1.53, *p* = 5.7E-06) were predictive for poor OS.

**MATERIALS AND METHODS:**

We integrated samples of three major cancer research centers (Berlin, Bethesda and Melbourne datasets) and publicly available datasets with available follow-up data to form a single integrated database. Subsequently, we performed a literature search for prognostic markers in gastric carcinomas (PubMed, 2012–2015) and re-validated their findings predicting first progression (FP) and overall survival (OS) using uni- and multivariate Cox proportional hazards regression analysis.

**Conclusions:**

The major advantage of our analysis is that we evaluated all genes in the same set of patients thereby making direct comparison of the markers feasible. The best performing genes include BIRC5, CASP3, CTNNB1, TIMP-1, MMP-2, SIRT, and VEGF.

## INTRODUCTION

Gastric cancer is one of the most common malignancies and displays variable incidence around the globe. About 90–95% of the cases are sporadic, it's incidence is highest in East Asia, Central Eastern Europe, and approximately 75% of cases present in less developed countries [1]. In most developed regions, rates of stomach cancer have variably but uniformly declined over the past decades, fairly due to active surveillance methods in selected societies [2]. There are no solid biomarkers besides HER2 [3] and regular clinicopathological parameters predicting prognosis and response to therapy, and ultimately, there are no efficient therapeutic options available which prove to change the outcome of patients in a groundbreaking manner.

Following endoscopic examination and histologic confirmation of malignancy in the harvested biopsy, the basis of therapy is still removal of the tumor mass utilizing surgery. The 5-year survival rate for R0 surgical resection ranges from 30 to 50% for patients with stage II and from 10 to 25% for patients with stage III disease [4]. As these patients have a high likelihood of local and systemic relapse, most centers offer them systemic therapy forming the other cornerstone of the treatment. In addition, radiation therapy has proved to improve 5-year survival in resectable tumors [5].

Applied prognostic factors of gastric cancer are limited to the clinicopathological properties in the routine setting today, and classically include the WHO histopathological type, Lauren-Järvi classification, size of the tumor, grade, invasion through the gastric wall (pT), vascular invasion, lymph node involvement (pN), etiological background (EBV or Helicobacter pylori) and HER2 overexpression [2, 5–7]. Biomarkers for diagnosis and prognosis of gastric cancer that have previously been identified are mostly non tumor tissue based, and include carcinoembryonic antigen (CEA), CA 125, CA 19–9, CA 72–4 and alpha-fetoprotein [6, 8], serum pepsinogen I, and proteases (pepsinogen C, plasminogen activator, matrix metalloproteinases and their inhibitors) [9]. Cadherins, mucins and CD44 splicing variants are related to invasion/metastasis and extracellular matrix adhesion and degradation [7].

Among tissue based markers, overexpression of human epidermal growth factor receptor 2 (HER2) has been identified as a negative prognostic factor [7]. Trastuzumab with chemotherapy in HER2-positive advanced gastric cancer was investigated in the ToGA study. In this phase 3 trial, 22% of advanced stage cancers overexpressed HER2 and overall survival with trastuzumab was 2.7 months longer (hazard ratio, HR = 0.74, *P* = 0.0046) [10]. In addition, trastuzumab improved all of the secondary end points as well.

In a search for robust cancer tissue related biomarkers, first we intended to perform a literature review and identify previously described markers for gastric cancer outcome. We merged transcriptomic data of multiple independent datasets to enable a cross-validation of these in a uniform independent cohort. We used uni- and multivariate analyses to assess the prognostic potential for each of the candidate markers. Finally, we compared expression in normal and gastric cancer samples to evaluate the change of the gene expression during tumor formation.

## RESULTS

### Database setup

The entire gastric cancer database includes 1,065 samples from seven independent datasets. Of these, 652 samples were measured with the Affymetrix Human Genome U133 Plus 2.0 Array, 145 with the Human Genome U133A 2.0 Array and 268 with the Human Genome U133A Array. Five arrays did not pass quality control and were excluded from the cross-validation analysis (all five arrays originated in the Bethesda dataset).

**Figure 1:**
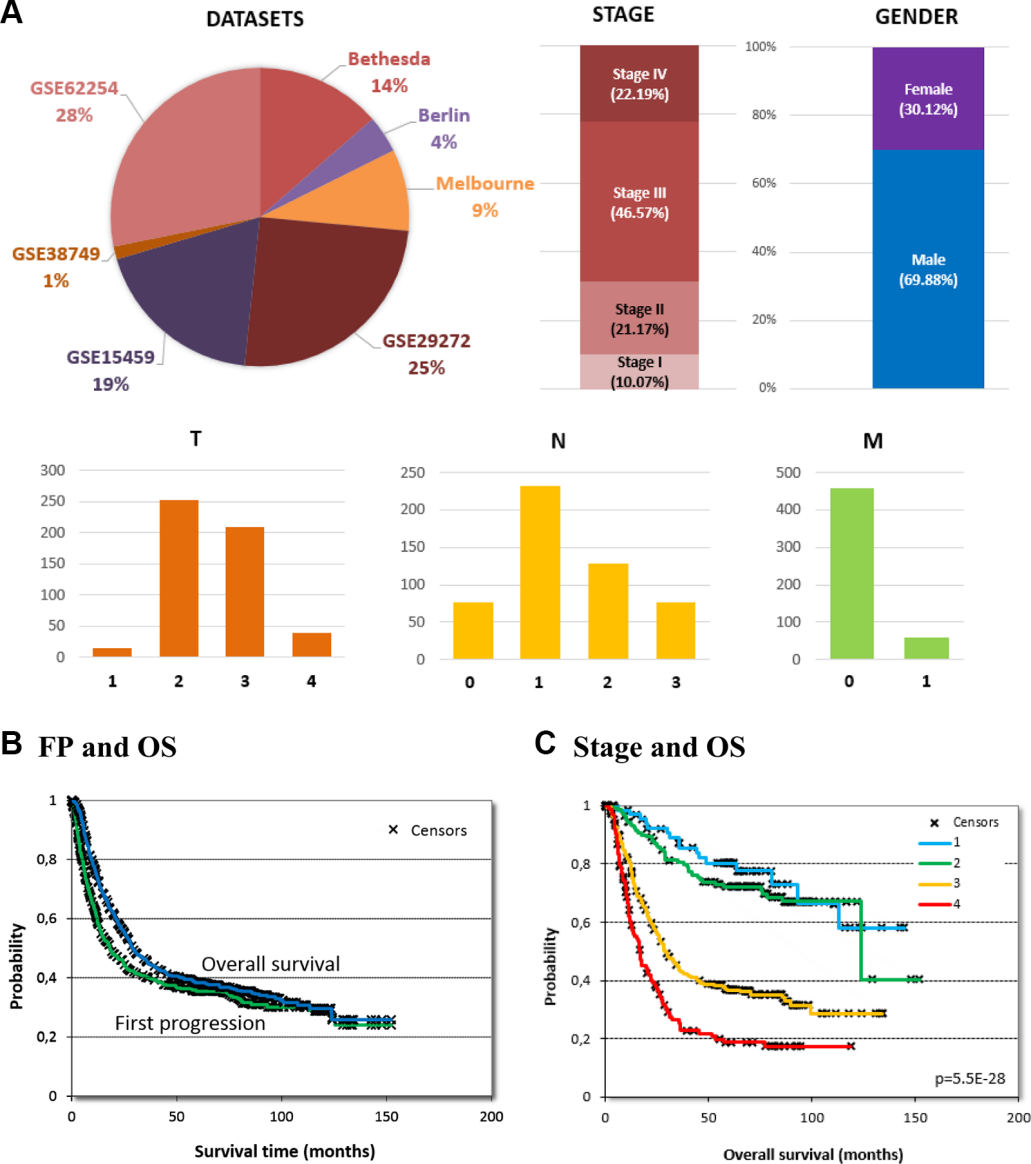
Database setup and clinical characteristics List of datasets included in the database as well as basic clinical characteristics **(A)**. Number of patients are given for TNM, because not all patients had these data available. Overall survival and time to first progression in all patients, **(B)** and effect of stage on overall survival **(C)**.

Gender and stage were available for most patients −70% of samples were male and stage III was most common (Figure 1A). Additional clinical parameters including TNM stages, histology and systemic treatment were available for about half the patients – the aggregate clinical characteristics are summarized in Table 1. The median time to first progression (FP) was 18.3 months and the median overall survival (OS) was 28.9 months. Even with these numerically significant differences, the survival curves comparing FP and OS display minor difference (Figure 1B) indicating a short post-progression survival – in the 503 patients with a first event and a known OS, the median post progression survival was 9.4 months.

**Table 1:** Summary of aggregate clinicopathological data for all patient samples included in the cross-validation

Parameter	%	N
**Gender**		
Male	53.1%	566
Female	22.9%	244
No data	23.9%	255
**Systemic treatment**		
No Adjuvant	36.9%	393
Adjuvant	22.3%	238
No data	40.8%	434
**Histology (Lauren class.)**		
Diffuse	23.3%	248
Intestinal	31.5%	336
Mixed	3.1%	33
No data	42.1%	448
**Differentiation**		
Poor	15.6%	166
Moderate	6.3%	67
Well	3.0%	32
No data	75.1%	800
**pT**		
T1	1.3%	14
T2	23.8%	253
T3	19.5%	208
T4	3.7%	39
No data	51.7%	551
**pN**		
N0	7.1%	76
N1	21.8%	232
N2	12.1%	129
N3	7.1%	76
No data	51.8%	552
**cM**		
M0	43.1%	459
M1	5.4%	58
No data	51.5%	548
**AJCC Stage**		
I	6.5%	69
II	13.6%	145
III	30.0%	319
IV	14.3%	152
No data	35.7%	380

Of the clinical parameters, gender, differentiation and histology were not significantly correlated to overall survival. Stage (*p* = 5.5E-28, see Figure 1C), T (*p* = 7.9E–15), and N (*p* = 1.1E-19) delivered high significance while there were not sufficient events to compute correlation to OS for M. Similar results were delivered for FP survival (stage: *p* = 1.7E-31, T: *p* = 9.2E-14, and N: *p* = 4.3E-20). In addition, M was also significant for FP (*p* = 1.3E-16).

### Identification of biomarker candidates

The keyword search in PubMed resulted in 775 hits, of which 749 were in English language, and 398 were published between 2012–2015. Of these, 40 publications were categorized as review. Following careful and critical evaluation, a list of 29 markers emerged (Supplementary Table 1). Of these candidates, one gene was not present on the gene chips (AFAP1L2), and the remaining 28 were evaluated in the cross-validation.

### Validation of previously identified prognostic markers

Out of the 28 biomarkers, 19 reached significance level with a FDR below 5% for FP and 20 for OS in the univariate analysis investigating gene expression only. Eighteen markers were significant for both FP and OS. Higher expression of BECN1, CASP3, COX2, CTGF, CTNNB1, MET, and SIRT1 correlated to better survival. Higher expression of BIRC5, CNTN1, EGFR, ERCC1, HER2, MMP2, PFKB4, SPHK, SP1, SPARC, TIMP1 and VEGF were predictive for poor outcome. For OS, the direction of correlation to survival was the same for all significant genes. The significant genes with hazard rates and *p* values are listed in Table 2. Supplementary Table 1. lists the results for all genes.

**Table 2:** List of significant gastric cancer genes evaluated in independent studies between 2012 and 2015

Symbol	Affy ID	Gene name	Ref.	First progression HR (95% CI), p	Overall survival HR (95% CI), p
BECN1	208946_s_at	Beclin-1	[44]	HR = 0.68 (0.55–0.84)*p* = 0.00042	HR = 0.68 (0.57–0.81) *p*= 1.5e-05
BIRC5	202094_at	Survivin	[25]	HR = 1.52 (1.22–1.89)*p* = 0.00016	**HR = 1.45 (1.2–1.75) *p*= 1e-04**
CASP3	202763_at	Caspase-3	[21]	HR = 0.52 (0.42–0.64)*p* = 3e-10	**HR = 0.5 (0.42–0.6) *p* = 6e-14**
CNTN1	211203_s_at	Contactin-1	[19]	HR = 1.41 (1.15–1.73)*p* = 0.0011	HR = 1.44 (1.21–1.7) *p* = 3.5e-05
COX2	204748_at	Cyclooxygenase-2	[16]	HR = 0.73 (0.59–0.91)*p* = 0.0056	HR = 0.72 (0.59–0.88) *p*= 0.0013
CTGF	209101_at	Connective tissue growth factor	[22]	HR = 0.71 (0.58–0.89*p* = 0.0022	HR = 0.72 (0.59–0.87) *p*= 0.00051
CTNNB1	201533_at	Beta-catenin	[18]	HR = 0.52 (0.42–0.64)*p* = 3.2e-10	**HR = 0.47 (0.38–0.57) *p*= 4.3e-15**
EGFR	201983_s_at	Epidermal growth factor receptor	[12]	HR = 1.85 (1.49–2.29)*p* = 1.6e-08	HR = 1.86 (1.54–2.25) *p*= 8.5e-11
ERCC1	203720_s_at	Excision repair complementation group 1	[45]	HR = 1.38 (1.12–1.69) p = 0.002	HR = 1.36 (1.13–1.63) *p*= 0.0012
HER2	216836_s_at	Human epidermal growth factor receptor 2	[46]	HR = 1.38 (1.12–1.69)*p* = 0.0021	**HR = 1.41 (1.18–1.68) *p*= 0.00011**
HIF1a	200989_at	Hypoxia-inducible factors-1 alpha	[14]	n.s.	HR = 0.73 (0.62–0.87) *p*= 0.00036
MET (HGFR)	203510_at	Hepatocyte growth factor receptor	[47]	HR = 0.69 (0.55–0.87)*p* = 0.0018	HR = 0.63 (0.51–0.77) *p*= 1.3e-05
MMP-2	201069_at	Matrix metalloproteinase 2	[24]	HR = 1.64 (1.33–2.02)*p* = 2.8e-06	HR = 1.78 (1.47–2.16) *p*= 2.6e-09
NOV	200724_at	Nephroblastoma Overexpressed	[22]	n.s.	HR = 1.45 (1.22–1.72) p = 1.7e-05
PFKB4	206246_at	6-phosphofructo-2-kinase/fructose-2,6-bisphosphatase-4	[48]	HR = 1.7 (1.33–2.19) *p* = 2.5e-05	HR = 1.56 (1.32–1.86) *p*= 3.2e-07
SIRT1	218878_s_at	Silent mating type information regulation 1	[49]	HR = 0.56 (0.45–0.7) *p* = 1.1e-07	**HR = 0.64 (0.54–0.76) *p*= 2.2e-07**
SPHK1	219257_s_at	Sphingosine kinase 1	[50]	HR = 1.62 (1.31–1.99)*p* = 5.6e-06	HR = 1.61 (1.31–1.96) *p*= 3.1e-06
SP1	214732_at	Specificity protein 1	[20]	HR = 1.47 (1.19–1.82)*p* = 4e-04	HR = 1.45 (1.23–1.72) *p*= 1.6e-05
SPARC	212667_at	Secreted protein acidic and rich in cysteine	[51]	HR = 1.34 (1.08–1.66)*p* = 0.007	n.s.
TIMP-1	201666_at	Tissue inhibitor of metalloproteinase-1	[23]	HR = 1.77 (1.42–2.22)*p* = 3.9e-07	**HR = 1.92 (1.57–2.36) *p*= 2.2e-10**
VEGF	210512_s_at	Vascular endothelial growth factor	[15]	HR = 1.75 (1.41–2.17)*p* = 2.9e-07	HR = 1.53 (1.27–1.85) *p*= 5.7e-06

**Figure 2:**
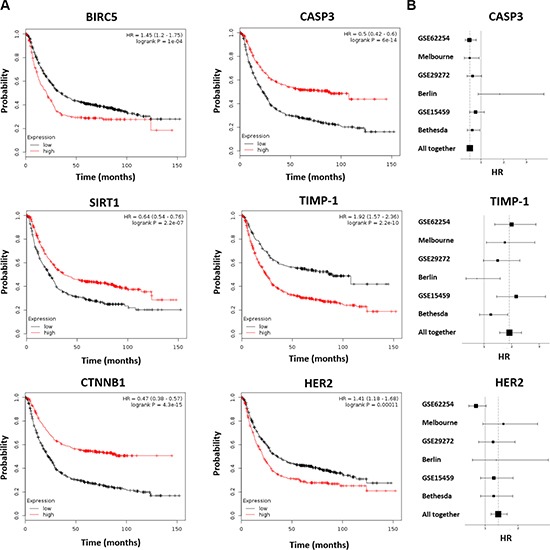
Survival for a selected set of the best performing markers Kaplan-Meier survival plots show that higher expression of CASP3, CTNNB1 and SIRT1 results in a better OS, while higher expression of BIRC5, TIMP-1 and HER2 lead to worse survival **(A)**. Forest plots for CASP3, TIMP-1, and HER2 **(B)**.

Statistical test: Cox univariate regression analysis, HR: hazard rate, CI: confidence interval, n.s.: *p* value over the 5% FDR cutoff. Bold: see survival plots in Figure 2.

Kaplan-Meier survival plots for three of the best performing genes with higher expression correlated to better survival (CASP3, SIRT1 and CTNNB1) and for three of the strongest genes with higher expression correlating to worse survival (BIRC5, HER2 and TIMP- 1) are presented in Figure 2.

### Multivariate analysis

When running a multivariate analysis for OS using all genes, BIRC5 (*p* = 0.0018), CASP3 (*p* = 2.3E-04), and CTNNB1 (*p* = 0.0011) were significant at *p* < 0.05. Marginal significance was achieved by SP1 (*p* = 0.07) and VEGF (*p* = 0.07). When also including stage in the multivariate model, only BIRC5 (*p* = 0.05) and stage (*p* = 1.1E-06) were significant. When including stage and age in the multivariate model, only HIF1alpha (*p* = 0.02), SPARC (*p* = 0.03), stage (*p* = 6.8E-07), and age (*p* = 0.002) were significant.

In case of FP, when including all genes in a multivariate Cox regression, BIRC5 (*p* = 0.0017), CASP3 (*p* = 9.7E-05), CTNNB1 (*p* = 0.01), MMP-2 (*p* = 0.0092), SIRT1 (*p* = 0.035), SPARC (*p* = 0.0024), and VEGF (*p* = 0.027) were significant at *p* < 0.05. However, when including stage or stage and age, only VEGF (*p* = 0.02), stage (*p* = 9.3E-07), and age (*p* = 0.01) remained significant. We have to note that the multivariate analysis used only a fraction of patients included in the univariate analysis, as not all patients had complete clinical annotation (*n* = 316 for OS and *n* = 240 for FP).

### Correlation to proliferation and HER2 expression

We used the expression of MKI67 as a surrogate of proliferation and run a Spearman correlation analysis for all genes. MKI67 itself had a strong prognostic value when examined in a univariate analysis for both overall survival (*p* = 0.0017, HR = 1.32) and relapse-free survival (*p* = 0.0015 and HR = 1.39).

Positive correlation to MKI67 expression was delivered by BIRC5 (coeff = 0.57, *p* < 1E-20), uPAR (coeff = 0.27, *p* = 3.9E-19), mTOR (coeff = 0.26, *p* = 1.6E- 18), SPHK1 (coeff = 0.21, *p* = 2.1E-12), and HER2 (coeff = 0.21, *p* = 2.8E-12). Negative correlation was observed for CTGF (coeff = −0.34, *p* = 9.6E- 31), SPARC (coeff = −0.31, *p* = 3E-25), PECAM-1 (coeff = −0.30, *p* = 9.2E-24), and SIRT1 (coeff = −0.23, *p* = 1.6E-14). As higher expression of multiple genes with negative correlation resulted in better survival (e.g. CTGF, SIRT1), and higher expression of genes with positive correlation delivered worse survival (e.g. BIRC5, HER2, SP1), we computed the correlation between the achieved hazard rate and the correlation coefficient against MKI67 expression. This analysis delivered a borderline significance (coeff = 0.32, *p* = 0.04). The same analysis performed for HER2 identified SP1 (coeff = 0.26, *p* = 5.1E-18), BIRC5 (coeff = 0.26, *p* = 5.2E-18), and EGFR (coeff = 0.20, *p* = 4.9E-17) having the highest correlation between gene expression and HER2 expression.

### Expression in non-tumor gastric tissues

The keywords “gastric” and “normal” GEO delivered 266 datasets. When reducing the search to individual platforms, nine datasets were generated with the GPL96, 35 datasets with the GPL570 and two datasets with the GPL571 platform. Of these, five datasets (GSE44740, GSE51725, GSE13911, GSE43346, and GSE3526) contained expression data for a total of 57 normal gastric tissue specimens.

**Figure 3:**
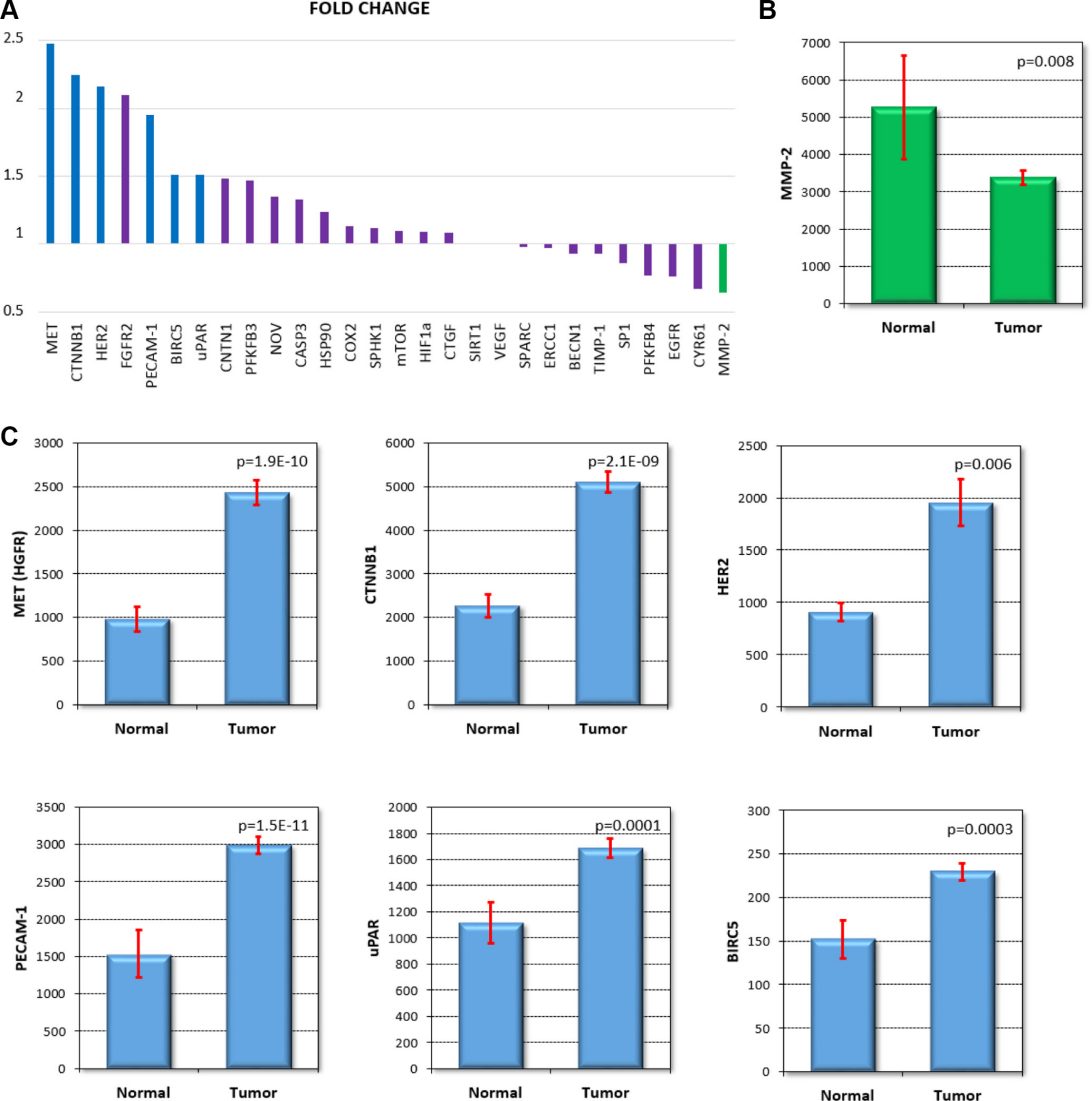
Expression change comparing normal and cancer tissue All markers ranked by the fold change **(A)**, MMP-2 was the only gene down regulated at *p* < 0.01 and FC < 0.66 **(B)**. Six genes had an expression increase over 1.5 fold with a *p* < 0.01 **(C)**. *The normalized expression values are shown for each gene. p: Mann-Whitney p* value *comparing normal and tumor samples. Red bar: 95% confidence interval*.

When comparing gastric normal and tumor samples, of all 28 genes, 6 were significant below *p* < 0.01 and had a fold change increase over 1.5 (BIRC5, CTNNB1, HER2, MET, PECAM-1 and uPAR) while only one gene had a 1.5-fold change reduction at the same significance (MMP- 2). The means with 95% confidence intervals for these genes are presented in Figure 3. Supplementary Table 2 contains all the expression values with the Mann-Whitney *p* value for each gene.

## DISCUSSION

In this project, we performed a validation for previously identified biomarkers for gastric cancer outcome and prognosis prediction. To assemble a sizeable patient number, we collected data from three cancer centers around the globe (Europe, USA, Australia), and integrated with additional publicly available datasets. Then, in a literature search we selected 28 relevant markers from the past few years (2012–2015), which were disclosed to be related to survival of gastric adenocarcinoma. Analysis was restricted to the most relevant genes by assessing only markers which had been previously published in review manuscripts.

Most of the molecules were related to worse outcome, being negative prognostic factors. The significant genes include members of the epidermal growth factor family and related genes (EGFR, HER2, SP1) [11–13], hypoxia-induced and angiogenic genes (HIF1A, VEGF, COX-2) [14–16], members of the MET and NOTCH signaling pathway (MET, CTNNB1, CNTN1) [17–19], regulators of survival and proliferation (SP1, CASP3, CTGF) [20–22], and genes involved in cellular motility (TIMP, MMP2) [23, 24]. All these genes are members of important pathways that contribute to progression of the neoplastic process through proliferation and survival by reprogramming the cell's metabolism, mitochondrial functions, protein and lipid synthesis, cytoskeletal organization and signaling.

The strongest candidate gene was BIRC5 (baculoviral inhibitor of apoptosis repeat containing 5, also known as survivin) – although numerically other genes (like TIMP-1) reached a higher hazard rate, but survivin remained significant in a multivariate analysis as well. BIRC5 has multiple effects including inhibition of apoptosis, enhancing cell proliferation, and promotion of angiogenesis [25]. The correlation between survivin and gastric cancer survival was described in multiple studies [25, 26]. In addition to effect on survival, we observed the highest correlation between BIRC5 and MKI67 expression which supports the link between survivin expression and progression through the cell cycle. However, MKI67 expression *per se* was not correlated to survival in our Cox regression analysis suggesting that the main effect of BIRC5 on survival is independent of cell proliferation. Theoretically, targeted therapy could be pursued in gastric cancer with siRNA, e.g. LY2181308, is investigated against survivin in multiple types of cancer, however outcome data is pending. Furthermore, immunotherapy may hold promise for these patients as survivin is a strong antigenic peptide for the T cells [27].

Among the most promising markers is SIRT1 (Silent mating type information regulation 1), a key gene in the progression of gastric cancer. Earlier, it's expression was associated with histological type, stage, lymph node status, and p53 expression [28], and proliferation as measured by Ki-67 index [29]. It was also identified as being down-regulated in gastric cancer and a key regulator of NFκB/Cyclin D1 signaling and G1 phase arrest [30], offering a possible therapeutic intervention in biological models. Here, our results confirm both the association between high SIRT1 and better survival and the correlation between SIRT1 and MKI67 expression. In theory, targeting SIRT1 can be utilized in cancer therapy, mostly cell cycle arrest in G1 phase showed promise in prostate cancer with DU145 [31].

Previously, receptor tyrosine kinase genomic alterations were detected in 20.6% of cases, affecting ERBB2, FGFR2, and MET, suggesting potential benefit from targeted therapy including MET-amplified gastric tumors and ERBB2 base substitutions [32]. Temporary but durable response to anti-MET agents have already been described [33]. Here, we observed a significant correlation between HER2 and MET and survival, but FGFR2 was not significant. Nevertheless, currently only one of the investigated genes, HER2 expression and amplification is utilized in the routine for prediction of response to anti-HER2 therapy [10].

Interestingly, the targetable genes (by administering e.g. cobimetinib, trastuzumab, and ponatinib, respectively, against) MET, HER2, and FGFR2 also displayed the highest difference with a fold change of the mean expressions over 2 when comparing gastric normal and cancer samples. However, again, FGFR2 was not significant – the reason for this is the uneven distribution of FGFR2 expression resulting in almost overlapping median expression at the same time.

Although our database represents a wide range of clinical cases, the patients are still not sufficiently characterized and this leads to a limitation of our study. While HER2 is an important marker also utilized clinically, our patient samples were collected before the introduction of anti-HER2 therapy. Thus, it was not possible to evaluate the effect of anti-HER2 therapy in the dataset. In the recent years, The Cancer Genome Atlas (TCGA) project proposed a molecular classification dividing gastric cancer into four subtypes [34]. We were also not able to validate the markers in these subtypes as the classification was not available for the patients included in the investigated datasets.

An alternative approach for survival prediction would be avoiding the utilization of a pre-defined gene to assign patients into prognostic cohorts. Rather, a whole transcriptome gene expression signature could be utilized to select molecularly similar patients and then determine prognostic expectations by evaluating the clinical outcome for these similar patients as has been demonstrated recently [35]. However, no similar methodology has been proposed for gastric cancer, thus we have not included such a model in our meta-analysis.

In summary, we collected gene expression data sets from three institutions and merged these with public datasets. Then, we performed a literature review and validated previously described markers for gastric cancer outcome. The major advantage of our analysis is that we evaluated all genes in the same set of patients thereby making direct comparison of the markers feasible. The best performing genes include BIRC5, CASP3, CTNNB1, TIMP-1, MMP-2, SIRT, SPARC, and VEGF. The importance of pathological parameters is supported by the fact that only a few genes remained significant when also including stage and age in a multivariate analysis.

## MATERIALS AND METHODS

### Identification of previously described biomarker candidates

We performed a literature search in PubMed (http://www.ncbi.nlm.nih.gov/pubmed) restricted to the timeframe of 2012 to 2015 utilizing the keywords “gastric”, “cancer”, “survival”, “gene expression” and “biomarker”. To limit the analysis to the most promising markers we selected only the English language manuscripts that were categorized as reviews. We performed the search in November/2015, and then manually continued with review of the publications one by one. Helicobacter pylori infection as a predisposing factor was not analyzed as a prognostic factor of outcome as there was no gene expression marker directly correlated to the infection. We assigned unique gene identifiers for each gene using the HUGO Gene Nomenclature Database (http://www.genenames.org/).

### Database setup

We assembled a gastric cancer database using samples measured in three different sources including previously partly published data at the Max Delbrück Center for Molecular Medicine, Berlin, Germany (“Berlin dataset”, published in GEO as GSE22377) [36]; at the Transgenic Oncogenesis and Genomics Section, Laboratory of Cancer Biology and Genetics, National Cancer Institute, Bethesda, Maryland, USA (“Bethesda dataset”, published in GEO as GSE14210) [37]; and at the Peter MacCallum Cancer Centre, Melbourne, Australia (“Melbourne dataset”, published in GEO as GSE51105) [38]. Sample collection, hybridization, and gene expression measurements were described previously. The clinical data was updated for each dataset at the end of 2014 and we utilized in the analysis the aggregate database containing all samples with available follow-up data.

### Publicly available datasets

We further extended the database using gene expression data downloaded from GEO. For this, we utilized the keywords “gastric”, “cancer”, “GPL96”, and “GPL570” to search GEO (http://www.ncbi.nlm.nih.gov/geo/). Only publications with available raw data, clinical survival information, and at least 15 patients were included. Affymetrix HG-U133A (GPL96) and HG-U133 Plus 2.0 (GPL570) microarrays were considered because of their overlapping set of 22,277 probe sets and because of our datasets were also derived using these gene chips.

### Database of normal gastric samples

To discriminate genes related to carcinogenesis, we assembled a database of normal tissues. For this, we used the keywords “gastric” and “normal” in GEO without any limitation regarding publication time or sample number within the study. We included only the GPL96, GPL570, and GPL571 platforms in the search. Samples with premalignant conditions such as intestinal metaplasia were not included as “normal”.

### Statistical analyses

The raw CEL files were MAS5 normalized in the R statistical environment (http://www.r-project.org) using the Affy Bioconductor library. Quality control for gene chips and control for duplicate samples were performed as described previously [39]. Only arrays passing the quality criteria were utilized. After normalization, only probes measured on both GPL96 and GPL570 were retained (*n* = 22,277). We subsequently performed a second scaling normalization to set the average expression on each chip to 1000 to reduce batch effects [40]. Kaplan–Meier survival plot and the hazard ratio with 95% confidence intervals and log-rank *P* values were calculated and plotted in R using Bioconductor packages. False discovery rate (FDR) was computed to correct for multiple testing using the brainwaver library in R as described previously [41] – the FDR cutoff was set at 5%. Expression in cancerous and normal samples was compared using a Mann-Whitney *U*-test.

### Multivariate analysis

We performed a multivariate analysis using Cox proportional hazards regression including the gene expression markers and clinical variables including stage, age, Lauren classification, differentiation, and gender. In addition to the clinical data, we also determined the HER2 and MKI67 expression using data provided on the gene chips. We computed HER2 status by using the probe set 216836_s_at and setting the cutoff for positivity at 4800 [42]. To assess correlation to proliferation, Spearman correlation to MKI67 expression (probes set 212021_s_at) was computed for each of the genes separately [43]. In addition, Spearman correlation was also run for HER2 without using the dichotomization.

## SUPPLEMENTARY MATERIALS FIGURES AND TABLES






