# Supplementary material for: Cross-validation of survival associated biomarkers in gastric cancer using transcriptomic data of 1,065 patients
Source: Oncotarget. 2016 Jun 30;7(31):49322–33. doi: 10.18632/oncotarget.10337 (PMC5226511; doi:10.18632/oncotarget.10337)
Supplement: Supplementary file 1 [file oncotarget-07-49322-s001.pdf]

Cross-validation of survival associated biomarkers in gastric cancer using transcriptomic data of 1,065 patients

Supplementary Materials

Supplementary Table S1: List of 29 biomarkers emerged in gastric cancer. See Supplementary_Table_S1

Supplementary Table S2: All the expression values with the Mann-Whitney *p* value for each gene, when comparing gastric normal and tumor samples. Of all 28 genes, 6 had a fold change increase over 1.5 (BIRC5, CTNNB1, HER2, MET, PECAM-1 and uPAR), one gene had a 1.5-fold change reduction (MMP-2). See Supplementary_Table_S2
